# Cellular responses to 8-methyl nonanoic acid, a degradation by-product of dihydrocapsaicin, in 3T3-L1 adipocytes

**DOI:** 10.1186/s12906-023-03844-w

**Published:** 2023-01-21

**Authors:** Uthai Wichai, Ploychanok Keawsomnuk, Saowarose Thongin, Chaiyot Mukthung, Chatchai Boonthip, Pattama Pittayakhajonwut, Pimonrat Ketsawatsomkron, Nuntavan Bunyapraphatsara, Kenjiro Muta

**Affiliations:** 1 Department of Chemistry and Center of Excellence in Biomaterials, Faculty of Science, Naresuan University, Phitsanulok, Thailand; 2 Chakri Naruebodindra Medical Institute, Faculty of Medicine Ramathibodi Hospital, Mahidol University, 111 Bang Pla, Bang Phli, Samut Prakan, 10540 Thailand; 3 Department of Chemistry, Faculty of Science, Naresuan University, Phitsanulok, Thailand; 4 National Center for Genetic Engineering and Biotechnology (BIOTEC), National Science and Technology Development Agency (NSTDA), Pathum Thani, Thailand; 5 Department of Pharmacognosy, Faculty of Pharmacy, Mahidol University, Bangkok, Thailand

**Keywords:** Capsaicinoids, 8-methyl nonanoic acid, Adipocytes, Metabolic function

## Abstract

**Background:**

Capsaicinoids, such as dihydrocapsaicin (DHC), exert the health-promoting effects of chili peppers on energy metabolism. The metabolic responses to capsaicinoids are primarily mediated through transient receptor potential cation channel subfamily V member 1 (TRPV1). However, the varying contributions of their metabolites to beneficial health outcomes remain unclear. 8-methyl nonanoic acid (8-MNA), a methyl-branched medium chain fatty acid (MCFA), is an in vivo degradation by-product of DHC. Since MCFAs have emerged as metabolic modulators in adipocytes, here we examined various cellular responses to 8-MNA in 3T3-L1 adipocytes.

**Methods:**

The viability of 3T3-L1 adipocytes exposed to various concentrations of 8-MNA was assessed by the Calcein AM assay. Biochemical assays for lipid accumulation, AMP-activated protein kinase (AMPK) activity, lipolysis and glucose uptake were performed in 3T3-L1 adipocytes treated with 8-MNA during 48-h nutrient starvation or 5-day maturation.

**Results:**

8-MNA caused no impact on cell viability. During nutrient starvation, 8-MNA decreased lipid amounts in association with AMPK activation, a molecular event that suppresses lipogenic processes. Moreover, 3T3-L1 adipocytes that were treated with 8-MNA during 5-day maturation exhibited a reduced lipolytic response to isoproterenol and an increased glucose uptake when stimulated with insulin.

**Conclusions:**

These results suggest that 8-MNA derived from DHC modulates energy metabolism in adipocytes and also support the idea that the metabolic benefits of chili consumption are partly attributable to 8-MNA.

**Supplementary Information:**

The online version contains supplementary material available at 10.1186/s12906-023-03844-w.

## Background

Consumption of chili peppers can modulate energy metabolism to induce numerous health benefits. The bioactive ingredients responsible for the spiciness and beneficial effects of chili peppers are capsaicinoids including capsaicin (CAP) and dihydrocapsaicin (DHC) [1, 2]. However, there are two major drawbacks to the therapeutic use of capsaicinoids that should be considered. Firstly, pharmacological doses of capsaicinoids cause overt discomfort in the gastrointestinal tract [3, 4], and secondly, capsaicinoids are rapidly degraded in the body. For instance, only 1.24 and 0.057% of ingested capsaicinoids remain in blood circulation 24- and 48-h after consumption, respectively. Therefore, studies that have measured the nutraceutical potential of capsaicinoids typically underrate the outcomes due to low bioavailability [5].

While the biological effects of capsaicinoids are mainly mediated via direct interaction with transient receptor potential cation channel subfamily V member 1 (TRPV1), other mechanisms were reported to be independent of this receptor [6]. The latter studies raise the possibility that capsaicinoid metabolites may be partly accountable for the overall biological outcomes of capsaicinoid intake. Indeed, an earlier metabolic tracer study demonstrated that 15% of DHC tracer was found as 8-methyl nonanoic acid (hereinafter referred to as 8-MNA) in the portal vein of rats, and the remaining radioactivity derived from tritiated DHC (85%). This is because of capsaicinoid hydrolysis activity in the jejunum, which is the location that ingested capsaicinoids are mainly absorbed [7]. Moreover, a previous in vitro study showed that in liver homogenates, the majority of DHC was metabolized into 8-MNA [8]. These findings demonstrate that 8-MNA is a ubiquitous degradation by-product of DHC. Aside from DHC, 8-MNA is a precursor material for CAP in chili fruits [9].

8-MNA is a methyl-branched derivative of nonanoic acid (C9:0) that is a unique odd-numbered medium chain fatty acid (MCFA) [10]. C9:0 was previously suggested to function as a selective activator of the peroxisome proliferator-activated receptor gamma (PPARγ), a transcriptional regulator of lipid and glucose metabolism [11, 12]. The potential health benefits of an MCFA-rich diet, especially octanoic acid (C8:0) and decanoic acid (C10:0), have been intensively studied in rodents and humans [13]. Consequently, these MCFAs have been discovered to be metabolic modulators in adipocytes [14, 15]. However, the cellular mechanisms that underlie the nutritional effects of 8-MNA have not been elucidated. Here, we hypothesized that 8-MNA, an in vivo metabolite of DHC, induces metabolic changes in adipocytes. To this end, we evaluated several cellular responses of 3T3-L1 adipocytes exposed to 8-MNA.

## Methods

### Chemical preparation

**Fig. 1:**
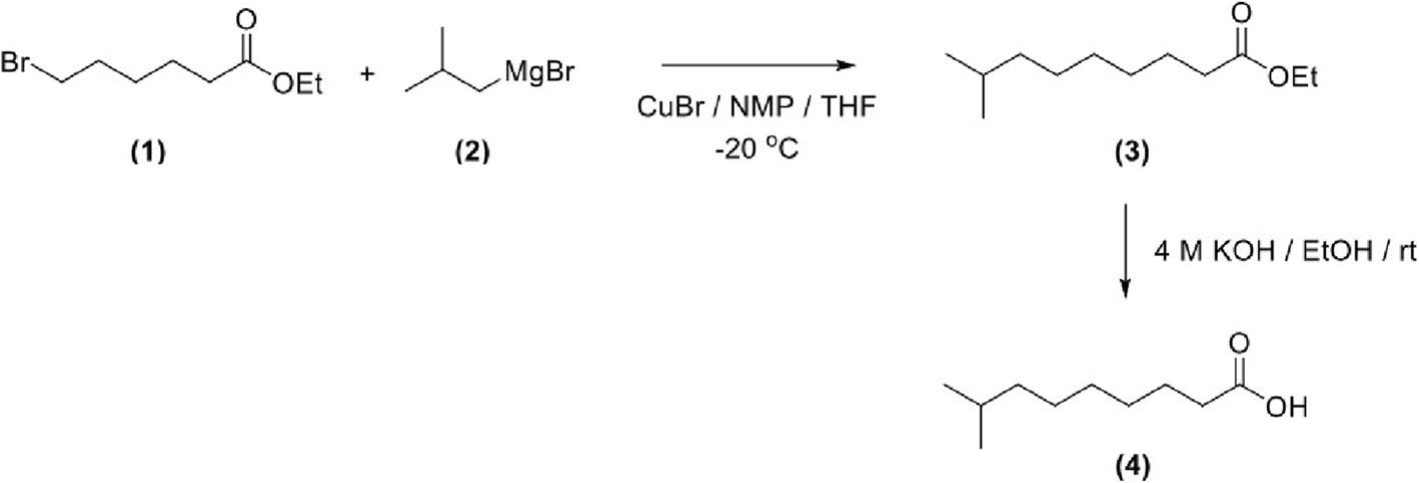
8-MNA synthesis. 8-MNA was synthesized via a copper-catalyzed alkylation of Grignard reagent, followed by hydrolysis of an ester under basic conditions. (1) ethyl-6-bromohexanoate, (2) isobutyl magnesium bromide, (3) ethyl 8-methylnonanoate, and (4) 8-methyl nonanoic acid

CAP and DHC were synthesized in our previous report [16]. 8-MNA was synthesized as shown in the diagram (Fig. 1). Copper(I) bromide (CuBr, 1.45 g, 10.08 mmol) and n-methyl-2-pyrrolidone (NMP, 129.7 mL) were dissolved in tetrahydrofuran (THF, 150 mL) and cooled in an ice-NaCl bath (3:1). Ethyl-6-bromohexanoate (**1**, 75.00 g, 336.16 mmol) was added dropwise by a cannula tubing. The solution of isobutyl magnesium bromide (**2**, 54.23 g, 336.16 mmol) was cooled in an ice-NaCl bath (3:1) and slowly added dropwise by a cannula tubing over 40 min, followed by stirring at room temperature overnight. The mixture was added to ammonium chloride solution (10%, 100 mL), and then it was extracted with hexane (2 × 100 mL). The organic layer was washed with ammonium chloride solution (10%, 200 mL). Finally, the solution was dried over anhydrous sodium sulfate, and the filtrate was concentrated under reduced pressure to yield ethyl 8-methylnonanoate (**3**) as a pale-yellow oil. The ester was dissolved in ethanol (125 mL), and aqueous KOH (4 M, 125 mL) was added dropwise at room temperature. After 90 min, the mixture was concentrated under reduced pressure to remove EtOH. The aqueous layer was acidified to a pH value of approximately 2–3 with 4 M HCl. The mixture was extracted with hexane (2 × 200 mL) and washed with water (200 mL). The organic layer was dried over anhydrous sodium sulfate and concentrated under reduced pressure to yield 8-methyl nonanoic acid (**4**) as a pale-yellow oil (55.00 g, 95% yield), R_f_ = 0.51 (25% EtOAc in Hexane); ^1^H NMR (400 MHz, CDCl_3_(d) ppm): 0.86 (d, *J* = 6.6 Hz, 6H), 1.11–1.19 (m, 2H), 1.22–1.38 (m, 6H), 1.51 (m, 1H), 1.58–1.68 (m, 2H), 2.34 (t, *J* = 7.5 Hz, 2H); FT-IR (ATR) (cm^−1^): 1705 (C = O stretching), 1288 (C-O stretching), 933 (O–H bending).

Human recombinant insulin (Novo Nordisk, USA) was diluted with sterile phosphate-buffered saline (PBS, pH 7.2, Gibco, USA) just before use. Otherwise, the chemicals were diluted in dimethyl sulfoxide (DMSO, Sigma-Aldrich, USA) and stored at -20 °C until use.

### Cell culture

The 3T3-L1 cells were purchased from American Type Culture Collection (CL-173, ATCC, USA). The cells were grown in Dulbecco’s Modified Eagle’s Medium (DMEM, Gibco, USA) supplemented with 10% bovine calf serum (ATCC, USA) in a humidified incubator with 5% CO_2_ at 37 °C, as recommended by the supplier.

### Short-term treatment of 3T3-L1 adipocytes with 8-MNA

Two days post confluency, the growth medium was replaced with DMEM containing 10% fetal bovine serum (FBS, Sigma-Aldrich, USA) and a cocktail of 1.5 µg/mL insulin, 1 µM dexamethasone, 500 µM 3-isobutyl-1-methylxanthine (IBMX), and 1 µM pioglitazone for differentiation. On Day 2, the differentiation medium was replaced with DMEM containing 10% FBS and 1.5 µg/mL insulin to promote maturation. The maturation medium was replaced every other day until Day 7, at which point the cells undergo a viability test and lipolysis assay (Protocol 1). Otherwise, on Day 4, the cells were incubated with serum-free DMEM containing DMSO (0.1%), 8-MNA (1 and/or 10 µM), CAP (1 µM), or DHC (1 µM) for 48 h for Oil Red O staining and western blotting.

### Cell viability assay

Cell viability was evaluated by quantifying the calcein AM permeability of intact cells [17]. The differentiated 3T3-L1 adipocytes in a 96-well black culture plate with a clear bottom were incubated with DMSO (0.1%) or various concentrations of 8-MNA (10 nM to 1 mM) in serum-free DMEM for 24 h and then treated with 1 µM calcein AM for 30 min. After washing excess calcein AM with PBS, the calcein AM fluorescence intensity was quantified using a fluorescence microplate reader.

### Oil red O (ORO) staining and quantification

Cells in a 24-well culture plate were washed with PBS twice and fixed with 4% paraformaldehyde (PFA, Wako, Japan) for 10 min. Fixed cells were washed with dH_2_O twice and incubated with 60% isopropanol for 5 min. After removing isopropanol, fixed cells were incubated with an ORO solution (Sigma-Aldrich, USA) for 20 min and then washed with dH_2_O three times. Images of the ORO-stained cells were captured with an inverted microscope. For quantification, ORO dye was extracted in 100% isopropanol for 5 min with gentle rocking. The absorbance of the extract was read at 492 nm using a spectrophotometer to quantitatively measure ORO staining of cellular neutral lipids.

### Western blotting

Cells in a 6-well culture plate were washed with PBS once and then lysed in chilled 50 mM Tris–Cl buffer (pH 7.5) containing 0.1 mM EDTA, 0.1 mM EGTA, 1% sodium deoxycholic acid (wt/vol), 1% NP-40 (vol/vol), 0.1% sodium dodecyl sulfate (vol/vol), 1 mM phenylmethanesulfonylfluoride (PMSF), and a cocktail of phosphatase and protease inhibitors (Cell Signaling, USA) on ice for 30 min with occasional hand-rocking. After centrifuging whole cell lysate at 14,000 × g for 20 min at 4 °C, the supernatant was collected and used to measure protein concentration with a DC protein assay kit (Bio-Rad, USA). Protein lysate was separated by SDS-PAGE and transferred to nitrocellulose or PVDF membranes. The membrane was blocked with 5% non-fat dry milk or BSA in Tris-buffered saline with 0.1% Tween 20 for 1 h and then cut into strips so as to include a protein of interest in the middle. The membrane strip was incubated with a diluted primary antibody [AMPKα, phospho-AMPKα (Thr172), or β-actin (Cell Signaling Technologies, USA)] in the blocking buffer at 4 °C overnight. The membrane strips were incubated in the blocking buffer containing anti-rabbit IgG conjugated with HRP (Cell Signaling Technologies, USA) for 1 h. Western ECL Substrate (Bio-Rad, USA) was used to visualize signals. The optical density of detected bands was quantified using ImageJ.

### Long-term treatment of 3T3-L1 adipocytes with 8-MNA

Two days post confluency, 3T3-L1 cells were differentiated into adipocytes with DMEM containing 10% FBS, dexamethasone, IBMX, and insulin for 2 days and then matured in DMEM containing 10% FBS, IBMX, and insulin in the presence of DMSO (0.1%), pioglitazone (1 µM), or 8-MNA (1 and 10 µM). The medium was replaced every other day until a lipolysis assay (Protocol 2) and glucose uptake study were performed at Day 7.

### Lipolysis assay

Protocol 1 (for Fig. 5a-b): To assess whether 8-MNA induces lipolysis, 3T3-L1 adipocytes in a 96-well culture plate were treated with either DMSO (0.1%), 8-MNA (10 μM), or isoproterenol (100 nM, a positive control for lipolysis) [18] for 3 h and 24 h. Protocol 2 (for Fig. 5c): To determine the extent of lipolytic response to a β-adrenergic agonist, 3T3-L1 adipocytes in a 96-well culture plate were incubated with vehicle (0.1% DMSO) or isoproterenol (100 nM) for 1 h. Using the conditioned medium into which glycerol was released, a lipolysis assay was performed according to the manufacturer’s protocol (Lipolysis Colorimetric Assay Kit, BioVision, USA).

### Glucose uptake assay

Cells in a 96-well culture plate were starved in serum-free DMEM for 2 h, then followed by incubating in Krebs Ringer Phosphate HEPES (KRPH) buffer containing 2% fatty acid-free BSA for 1 h. A glucose uptake assay was performed using a colorimetric assay kit (BioVision, USA) 30 min after adding 1 mM 2-deoxyglucose (2-DG) with PBS or 0.1 µM insulin.

### Statistical analysis

The data were analyzed with Prism 8 (GraphPad, USA) and expressed as the mean ± SEM. Student’s t-test, one-way, or two-way ANOVA was used to compare the effect of various treatments. The results were considered statistically significant when the probability value was less than 0.05 (indicated as *p* < 0.05).

## Results

### Viability of 3T3-L1 adipocytes treated with 8-MNA

**Fig. 2:**
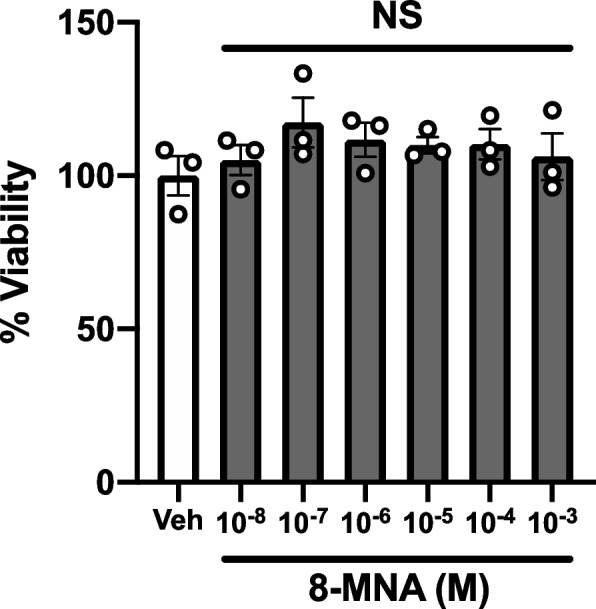
Effect of 8-MNA on the viability of 3T3-L1 adipocytes. Cells were treated with vehicle (Veh) or various concentrations of 8-MNA ranging from 10 nM to 1 mM for 24 h. Cell viability was determined by calcein AM assay. Data are presented as mean ± SEM and analyzed by one-way ANOVA (*n* = 3). NS, not significant vs. Veh. n, sample size

In contrast to the long-chain fatty acids (LCFAs)-induced cytotoxicity observed in 3T3-L1 cells [19], we hypothesized that 8-MNA would have no overt toxicity due to its structural resemblance to C9:0, which has been shown to be of low toxicity in rodents [10]. However, we still assessed the viability of 3T3-L1 adipocytes exposed to various concentrations of 8-MNA for 24 h. The viability of vehicle-treated adipocytes was set to 100% to compare with that of the 8-MNA-treated groups. Up to 1 mM, 8-MNA caused no harm to the viability of 3T3-L1 adipocytes as compared to vehicle (0.1% DMSO) (Fig. 2). For the following experiments, we selected two concentrations of 8-MNA (1 and/or 10 µM) equivalent to those concentrations of capsaicinoids used in previous studies examining lipid metabolism in adipocytes [16, 20].

### Effect of 8-MNA on fat accumulation in 3T3-L1 adipocytes

**Fig. 3:**
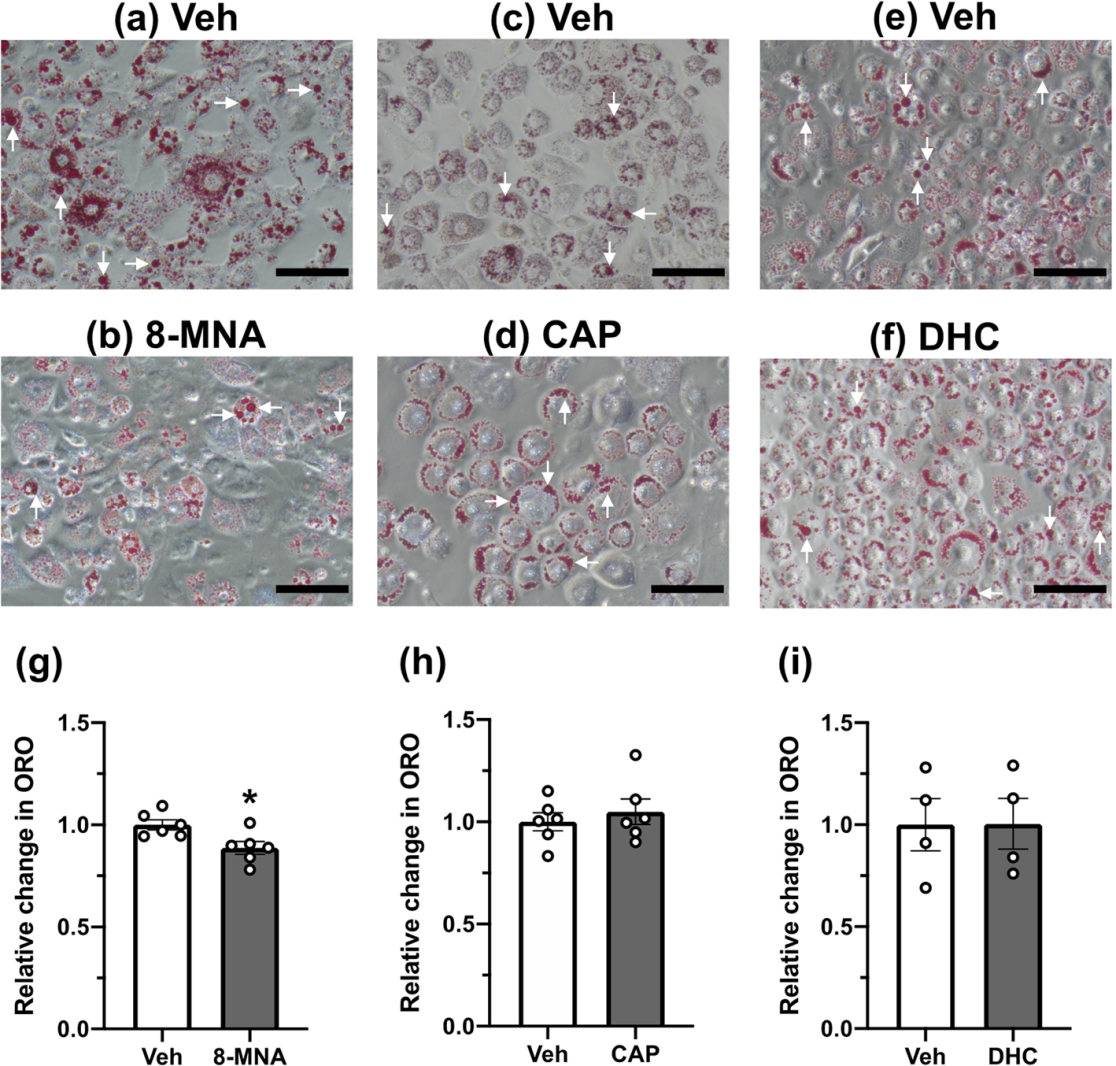
Effect of 8-MNA and capsaicinoids on fat accumulation in 3T3-L1 adipocytes. Representative images (**a**-**f**) and quantification (**g**-**i**) of ORO staining in 3T3-L1 adipocytes cultured with vehicle (Veh), 8-MNA (1 μM), CAP (1 μM), or DHC (1 μM) during 48 h starvation. Bright-field images were taken at 20X magnification. White arrows indicate representative lipid droplets. Scale bar = 100 μm. Data are presented as mean ± SEM and analyzed by Student’s t-test (n = 4–6). * *p* < 0.05 vs. Veh. n, sample size

To test the hypothesis that the biochemical activities of 8-MNA on adipocytes partly contribute to the prominent anti-lipogenic property of capsaicinoids in vivo [5], we examined the effect of 8-MNA on fat accumulation. The 3T3-L1 adipocytes were treated with vehicle (0.1% DMSO), 8-MNA, CAP, or DHC (1 µM for each) for 48 h in a serum-free medium. Neutral lipids stored in adipocytes were visualized by ORO staining (Fig. 3a-f) and subsequently quantified by measuring the absorbance of ORO dye eluents (Fig. 3g-i). The difference in the extent of ORO-stained lipid droplets between vehicle- and 8-MNA-treated groups was hardly distinguishable (Fig. 3a and 3b). However, ORO-based quantification detected a modest but significant decrease (-11%) in fat deposits of 3T3-L1 adipocytes exposed to 8-MNA relative to that of the vehicle group (Fig. 3g). To compare this lipid-lowering effect of 8-MNA with capsaicinoids, we repeated the ORO experiments under the same condition in the presence of CAP or DHC (Fig. 3c-f). Interestingly, neither CAP (Fig. 3h) nor DHC (Fig. 3i) at concentrations of 1 µM affected lipid accumulation. These results suggest that under the current experimental condition, 8-MNA functions as an anti-lipogenic factor, while CAP and DHC have no influence on overall lipid metabolism in 3T3-L1 adipocytes.

In addition, we explored the possibility that the differentiation of 3T3-L1 cells into adipocytes might be affected by 8-MNA through interfering with PPARγ pathway, a key transcriptional regulator for adipocyte differentiation [12, 21] (Supplementary Fig. 1). Treating 3T3-L1 cells with pioglitazone, a PPARγ agonist during differentiation significantly increased the extent of ORO staining relative to the control group exposed to neither pioglitazone nor 8-MNA. However, 8-MNA treatment concurrently with pioglitazone did not influence the pioglitazone-enhanced fat accumulation. We thus conclude that 8-MNA is incapable of affecting PPARγ pathway in the process of 3T3-L1 differentiation.

### Effect of 8-MNA on AMPK activity in 3T3-L1 adipocytes

**Fig. 4:**
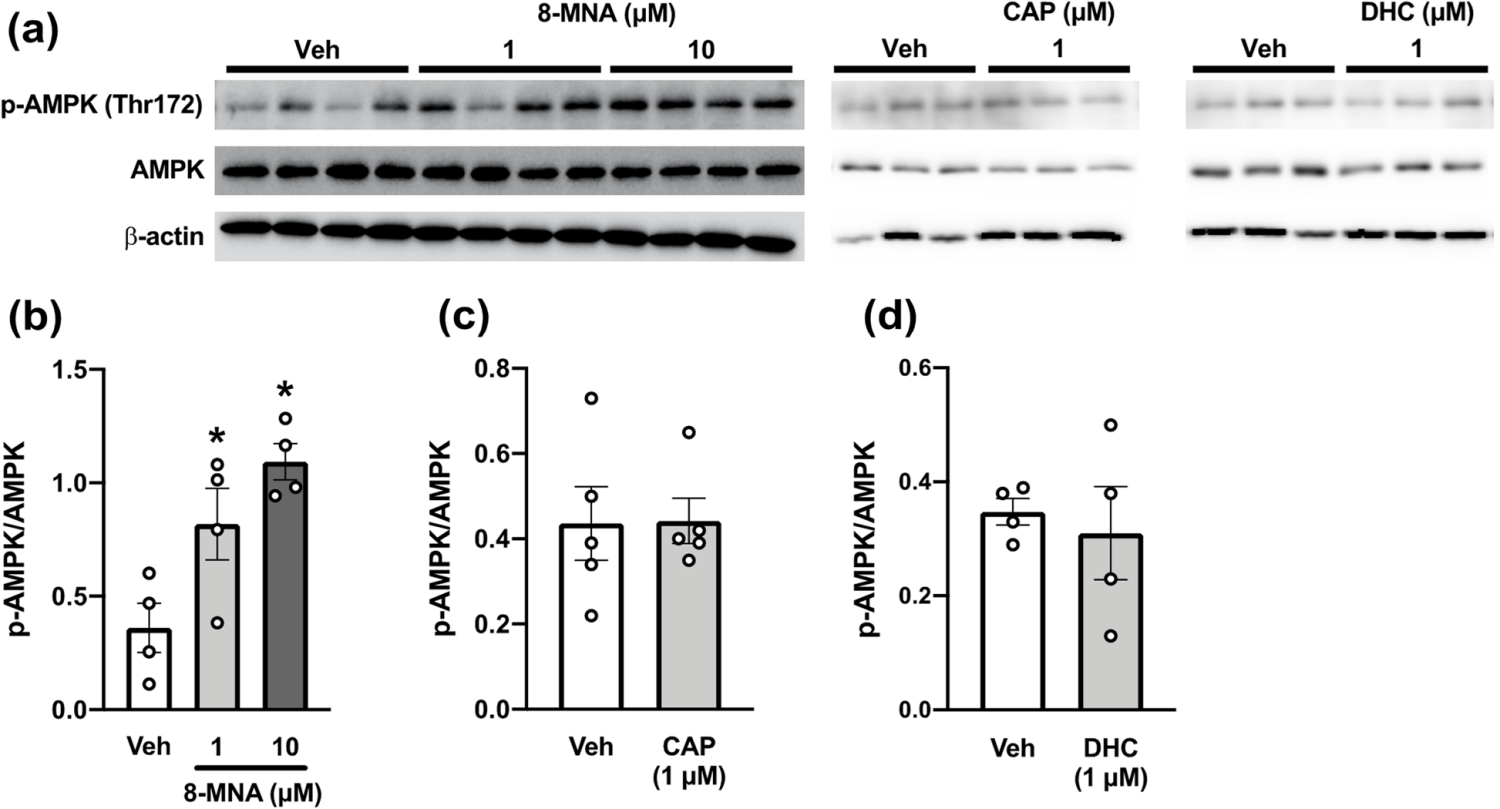
Effect of 8-MNA and capsaicinoids on AMPK activity in 3T3-L1 adipocytes. Western blotting (**a**) and quantification (**b**-**d**) of cell lysates collected from 3T3-L1 adipocytes cultured with vehicle (Veh), 8-MNA (1 and 10 μM), CAP (1 μM) or DHC (1 μM) during 48 h starvation. Data are presented as mean ± SEM and analyzed by one-way ANOVA in (**b**) (*n* = 4) and by Student’s t-test in (**c** and **d**) (*n* = 4–5). * *p* < 0.05 vs. Veh. *n*, sample size. The original blots are presented in Supplementary Figure

As we observed 8-MNA-mediated lipid reduction in adipocytes cultured in a serum-free environment (i.e., fatty acid-free, except for 8-MNA per se), 8-MNA potentially blocked de novo lipogenesis, a metabolic pathway that converts excess carbohydrates and amino acids into fatty acids for triacylglycerol synthesis. This process is inhibited by AMP-activated protein kinase (AMPK) [22]. Thus, AMPK pathway might be involved in the lipid-lowering effect of 8-MNA. To explore this possibility, 3T3-L1 adipocytes were treated with either vehicle or 1 or 10 µM 8-MNA for 48 h in the same manner as Fig. 3. Levels of AMPK phosphorylation at Thr172, which is required for its activation [22], were concentration-dependently increased by 8-MNA treatment (Fig. 4a-b). Next, 3T3-L1 adipocytes were stimulated with 1 µM CAP or DHC. Neither capsaicinoid increased AMPK phosphorylation (Fig. 4a, 4c, and 4d), which is in agreement with the effects we observed on fat accumulation (Fig. 3c-f and 3h-i). These results suggest that the suppression of de novo lipogenesis by 8-MNA is correlated with AMPK activation in 3T3-L1 adipocytes.

### Effect of 8-MNA on lipolysis in 3T3-L1 adipocytes

**Fig. 5:**
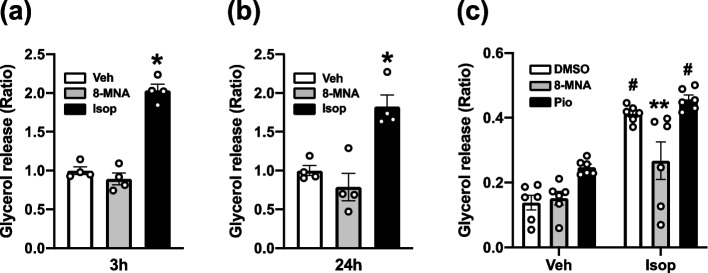
Effect of 8-MNA on lipolysis in 3T3-L1 adipocytes. (**a**-**b**) Cells were treated with vehicle (Veh), 8-MNA (10 μM), or isoproterenol (Isop, 100 nM) for 3 h (**a**) and 24 h (**b**). (**c**) Cells matured in the presence of DMSO (0.1%), 8-MNA (1 μM) or pioglitazone (Pio, 1 μM) for 5 days were stimulated with vehicle (Veh) or isoproterenol (Isop, 100 nM) for 1 h. Lipolysis was measured by quantifying a lipolytic end product, glycerol released into assay buffer. Data are presented as mean ± SEM and analyzed by one-way ANOVA in (a) and (b) (*n* = 4) and by two-way ANOVA in (c) (*n* = 6). * *p* < 0.05 vs. Veh and 8-MNA. ** *p* < 0.05 vs. Isop-DMSO and Isop-Pio. # *p* < 0.05 vs. Veh-treated groups. *n*, sample size

CAP has been shown to directly induce a lipolytic response in adipocytes [23], whereas MCFAs have the opposite effect when exposed to a β-agonist [24]. Therefore, we assessed the extent to which 8-MNA influences the lipolytic capacity of 3T3-L1 adipocytes, as indicated by the released amount of glycerol. 3T3-L1 adipocytes were treated with vehicle (0.1% DMSO), 8-MNA (10 µM), or isoproterenol (100 nM) for 3 and 24 h. As previously reported [18], isoproterenol treatment induced glycerol release relative to vehicle treatment (Fig. 5a-b). However, at both time points, 8-MNA treatments caused no change in glycerol release (Fig. 5a-b). Next, differentiated 3T3-L1 cells were incubated with DMSO (0.1%), 8-MNA (1 µM), or pioglitazone (Pio, 1 µM) for 5 days during maturation. At baseline, none of the treatments yielded a significant change in lipolytic capacity (Fig. 5c, Veh). Isoproterenol treatment for 1 h significantly increased glycerol release in DMSO- and pioglitazone-treated 3T3-L1 adipocytes in comparison to vehicle-treated counterparts (Fig. 5c, Veh vs. Isop). Conversely, the lipolytic effect of isoproterenol was significantly reduced by 8-MNA (Fig. 5c, Veh vs. Isop). These data suggest that 8-MNA is unable to directly influence lipolysis at baseline but the isoproterenol-induced lipolysis is attenuated in 3T3-L1 adipocytes chronically treated with 8-MNA.

### Effect of 8-MNA on glucose uptake in 3T3-L1 adipocytes

**Fig. 6:**
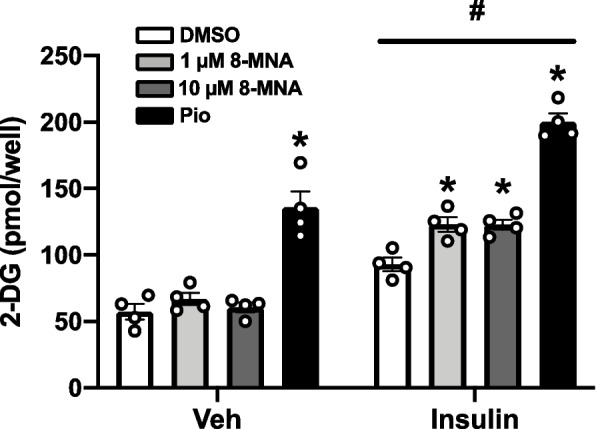
Effect of 8-MNA on glucose uptake in 3T3-L1 adipocytes. Cells matured in the presence of DMSO (0.1%), 8-MNA (1 and 10 μM), or pioglitazone (Pio, 1 μM) for 5 days were incubated in KRPH buffer containing 2-deoxyglucose (2-DG) in combination with vehicle (Veh) or insulin (0.1 μM) for 30 min. Data are presented as mean ± SEM and analyzed by two-way ANOVA (*n* = 4). * *p* < 0.05 vs. DMSO controls. # *p* < 0.05 vs. Veh-treated each group. *n*, sample size

MCFA supplementation to a high-fat diet given to rats has been reported to increase glucose disposal during glucose tolerance tests [25–27]. These studies suggested that 8-MNA could play a role in glucose metabolism. To determine whether 8-MNA affects glucose uptake, 3T3-L1 adipocytes were prepared in the same way as Fig. 5c and subsequently treated with vehicle (PBS) or insulin (0.1 µM) for 30 min in the presence of 2-DG, a non-metabolizable glucose analog. A PPARγ agonist, pioglitazone was used as a positive control since glitazone treatment of 3T3-L1 adipocytes enhances glucose uptake both at rest and in response to insulin [28]. In accordance with previous data, there was a significant increase in glucose uptake into 3T3-L1 adipocytes following pioglitazone treatment, even without insulin-mediated effects (i.e., GLUT4 translocation to the membrane), relative to vehicle control (Fig. 6, Veh). In contrast, neither 1 nor 10 µM 8-MNA changed the extent of basal 2-DG uptake (Fig. 6, Veh). When stimulated with 0.1 µM insulin, pioglitazone-treated 3T3-L1 cells exhibited a remarkable increase in 2-DG uptake relative to insulin-treated DMSO (Fig. 6, Insulin). Importantly, in response to insulin, 2-DG uptake into 8-MNA-treated cells at 1 and 10 µM significantly increased by ~ 25% as compared to that of the DMSO group (Fig. 6, Insulin). These findings led us to conclude that 8-MNA increased the sensitivity of 3T3-L1 cells to insulin, thereby enhancing glucose uptake.

## Discussion

The current study demonstrated that 8-MNA stimulates several metabolic responses in 3T3-L1 adipocytes. We observed that 8-MNA 1) produced no overt toxic effect, 2) reduced de novo lipogenesis which was correlated with concentration-dependent increases in AMPK activity, 3) decreased isoproterenol-induced lipolysis, and 4) increased glucose uptake in response to insulin.

Capsaicinoids are secondary metabolites in chili peppers and considered to be a potential therapeutic agent for obesity, cancer, and pain management. These therapeutic effects have been proposed to be mediated through either TRPV1 [29] or TRPV1-independent processes [6]. However, the pungent flavor and short bioavailability of capsaicinoids have hampered the feasibility of using them for medicinal purposes [5]. In previous studies, 8-MNA was found in portal blood of rats that had received tritiated DHC [7], as well as in cell-free liver homogenates mixed with DHC [8], suggesting that 8-MNA is an in vivo degradation product of DHC. These lines of evidence prompted us to explore the metabolic functions of 8-MNA.

Given that 8-MNA lacks the vanillyl head group of capsaicinoids that forms the hydrogen bond with TRPV1, 8-MNA effects unlikely resulted from TRPV1-dependent cell death [30]. Furthermore, while excessive LCFAs can reduce the viability of adipocytes [19], MCFAs, like 8-MNA, have been reported to cause no adverse effects [31], except for a marginal case in which MCFA-fed mice presented with myocardial oxidative stress and atrophy [32]. Nonetheless, to the best of our knowledge, 8-MNA toxicity has not been reported [10]. Predictably, 8-MNA-treated adipocytes showed no signs of cytotoxicity up to 1 mM, at which LCFAs decreased the viability of adipocytes to less than 50% [19]. However, further research on in vivo toxicity of 8-MNA is warranted to definitively extrapolate its nutraceutical usefulness.

Dietary intake of capsaicinoids has been shown to improve metabolic status by mainly increasing the release of catecholamines from the adrenal gland, where TRPV1 receptors are abundantly expressed [33]. This interaction results in heat generation (in skeletal muscles and brown adipose tissue) fueled by active lipid mobilization in white adipose tissue [34]. As reported by others [20, 35] and us [16], in addition to the systemic effects, capsaicinoids appear to act directly on adipocytes by decreasing fat accumulation. The nutritional and physiological outcomes of MCFAs have been intensively studied in rodents and humans undergoing obesogenic diet treatment with MCFA supplementation (or isocaloric replacement of LCFAs with MCFAs). These studies highlighted that MCFAs inhibit not only excess fat accumulation in white adipose tissues [36–38] but also ectopic fat accumulation in liver tissue [38, 39] and skeletal muscles [40]. Conversely, MCFA-mediated effects on lipid accumulation were unclear in in vitro studies. C8:0 and C10:0 MCFAs reduced lipid accumulation in 3T3-L1 preadipocytes during maturation with a hormonal cocktail [14], while other studies concluded that MCFAs actually increased fat content in hepatocytes [14, 41, 42]. Our findings here showed that under serum-free conditions, de novo lipogenesis in 3T3-L1 adipocytes was inhibited by 8-MNA but not by CAP or DHC. Thus, the effects of capsaicinoids on obesity in vivo are partly attributable to their metabolite, 8-MNA.

The AMPK pathway plays an important role in cellular energy metabolism and is activated during energy-poor states (i.e., increased AMP/ATP ratio) [43]. Particularly, AMPK activation inhibits de novo synthesis of triacylglycerol, a process that converts excess carbohydrates and amino acids into lipid storage, while promoting β-oxidation [44, 45]. Several reports have demonstrated that CAP causes AMPK activation, resulting in a reduction in lipid content in hepatocytes and adipose tissues [46, 47]. Similarly, MCFA-mediated AMPK activation in vivo has been documented in liver [48], skeletal muscle [49], and adipose tissues [50]. However, the influence of MCFAs on AMPK activity in vitro is still contentious [51]. Here, we found that in a serum-free environment, 8-MNA activated the AMPK pathway. In a recent report, in L6 myotubes, this MCFA-induced AMPK activation was shown to be mediated via a Ca^2+^/CaM-dependent kinase cascade [52]. Since MCFAs rapidly undergo β-oxidation after entering mitochondria, AMPK activation by 8-MNA may be a necessary process for energy production in starved cells. Accordingly, in adipocytes, the AMPK-inhibition of de novo lipogenesis resulted in reduced fat storage.

Although the lipolytic effect of capsaicinoids is mediated mainly through its action on adrenal glands, which induces secretion of catecholamines that activate lipolysis in adipose tissues [34], CAP has been reported to directly stimulate lipolysis in adipocytes [23]. In our study, which had a comparable experimental design to this report, we found that neither 3 h nor 24 h treatments of adipocytes with 8-MNA caused lipolysis. In contrast, prior findings that glitazone and MCFAs can suppress β-agonist-induced lipolysis [24, 53] were recapitulated here. In 3T3-L1 adipocytes matured with 8-MNA, we demonstrated an attenuation of lipolysis in response to isoproterenol, suggesting that this anti-lipolytic action of 8-MNA may contribute to the reduced amount of blood non-esterified fatty acids. Consequently, it may block ectopic fat accumulation in other metabolic tissues, such as liver tissue (i.e., steatosis) [53].

The process of adipocyte lipolysis is partly regulated by altering cellular cAMP concentrations in response to lipolytic agents and hormones [54, 55]. For instance, isoproterenol stimulates cAMP production to accelerate lipolysis, whereas insulin-mediated activation of phosphodiesterase 3B (PDE3B) degrades cAMP to inhibit lipolysis [54, 55]. In Fig. 6, we found the ability of 8-MNA to enhance insulin sensitivity in 3T3-L1 adipocytes, which was demonstrated by an increase in insulin-dependent glucose uptake. This result led us to speculate that 8-MNA possibly suppressed isoproterenol-induced lipolysis by activating PDE3B.

Effects of 8-MNA on glucose metabolism were proposed based on multiple reports. The anti-lipogenic effect of MCFA likely accounts for preventing the development of prediabetes by improving glycemic control [25–27]. Moreover, a previous in vivo study showed that intravenous C9:0 infusion for an hour caused a marked increase in insulin secretion in anesthetized dogs, followed by a corresponding reduction in blood glucose levels. Although the plasma levels of insulin returned to the pretreatment levels within 3 h post infusion, the blood glucose levels were remained low, suggesting that C9:0 administration could increase insulin sensitivity in the peripheral tissues [56]. Consistent with this report, in the current study, 8-MNA enhanced glucose uptake in an insulin-dependent manner, suggesting sensitization of insulin receptor signaling by 8-MNA.

## Conclusions

In conclusion, our findings demonstrate that 8-MNA exerts metabolic effects in adipocytes by decreasing de novo lipogenesis and lipolytic response to isoproterenol and increasing insulin-dependent glucose uptake. Since 8-MNA is an in vivo metabolite of capsaicinoids [7], the metabolic effects of chili consumption is likely due to both capsaicinoids and the 8-MNA metabolite. However, unlike capsaicinoids, 8-MNA is not pungent or cytotoxic, making it attractive for use as nutraceuticals in humans. To further determine physiologically-relevant responses to 8-MNA, animal studies are required to assess the extent to which 8-MNA supplement to obesogenic diets influences obesity and prediabetes. This approach could be warranted to answer questions regarding the systemic effects of 8-MNA.

## Supplementary Information

**Additional file 1:** **Supplementary Figure 1.** Effect of 8-MNA during differentiation on fat accumulation in 3T3-L1 cells. **Supplementary Figure 2.** The uncropped images of Figure 4a.

## Data Availability

All data generated or analyzed during this study are included in this published article and its supplementary information files.

## Abbreviations

2-DG: 2-Deoxyglucose
8-MNA: 8-Methyl nonanoic acid
AMPK: AMP-activated protein kinase
CAP: Capsaicin
C8:0: : Octanoic acid
C9:0: : Nonanoic acid
C10:0: : Decanoic acid
CuBr: Copper(I) bromide
DHC: Dihydrocapsaicin
DMEM: Dulbecco’s modified eagle medium
DMSO: Dimethyl sulfoxide
FBS: Fetal bovine serum
IBMX: 3-Isobutyl-1-methylxanthine
LCFA: Long-chain fatty acid
MCFA: Medium-chain fatty acid
NMP: N-methyl-2-pyrrolidone
ORO: Oil Red O
PBS: Phosphate-buffered saline
PPARγ: Peroxisome proliferator-activated receptor gamma
THF: Tetrahydrofuran
TRPV1: Transient receptor potential cation channel subfamily V member 1
