# Supplementary material for: Cellular responses to 8-methyl nonanoic acid, a degradation by-product of dihydrocapsaicin, in 3T3-L1 adipocytes
Source: BMC Complement Med Ther. 2023 Jan 21;23:18. doi: 10.1186/s12906-023-03844-w (PMC9862568; doi:10.1186/s12906-023-03844-w)
Supplement: Supplementary file 1 — Additional file 1: Supplementary Figure 1. Effect of 8-MNA during differentiation on fat accumulation in 3T3-L1 cells. Supplementary Figure 2. The uncropped images of Figure 4a. [file 12906_2023_3844_MOESM1_ESM.pdf]

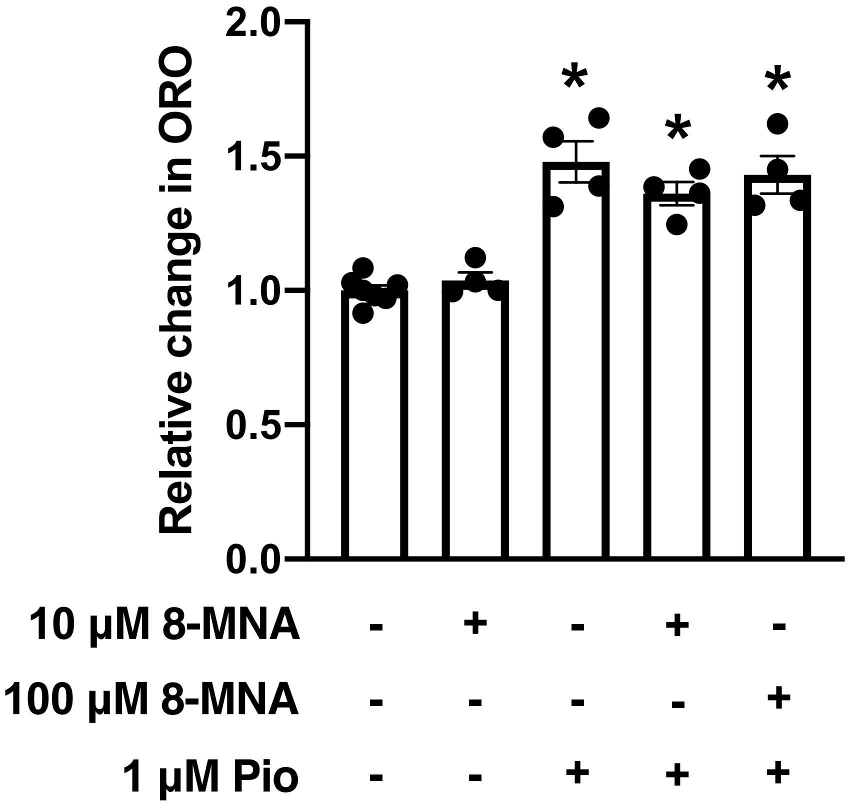

Supplementary Figure 1. Effect of 8-MNA during differentiation on fat accumulation in 3T3-L1 cells

Cells were differentiated for 48h in the presence of vehicle (0.1%DMSO), 8-MNA (10 or 100 µM) and/or pioglitazone (Pio, 1 µM), followed by 4-day culturing in the maintenance medium. Fat accumulation was quantified by measuring the absorbance of Oil Red O (ORO) eluent at 492 nm. Data are presented as mean ± SEM and analyzed by one-way ANOVA (n=4-7). * $p<0.05$ vs. Veh. n, sample size.

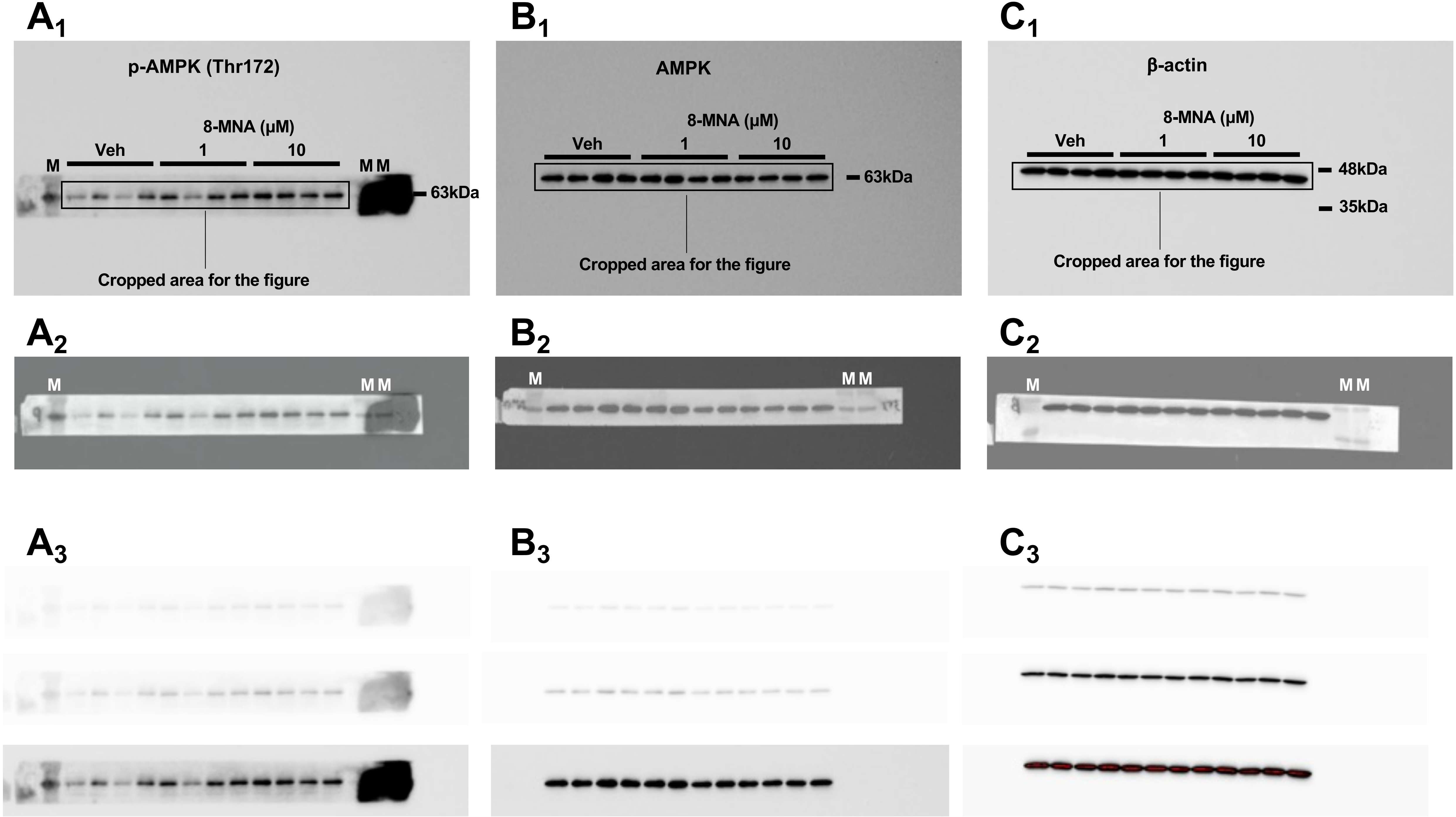
A1
p-AMPK (Thr172)
8-MNA (μM)
Veh
1
10
M
M M
63kDa
Cropped area for the figure
B1
AMPK
8-MNA (μM)
Veh
1
10
63kDa
Cropped area for the figure
C1
β-actin
8-MNA (μM)
Veh
1
10
48kDa
35kDa
Cropped area for the figure
A2
M
M M
B2
M
M M
C2
M
M M
A3
B3
C3

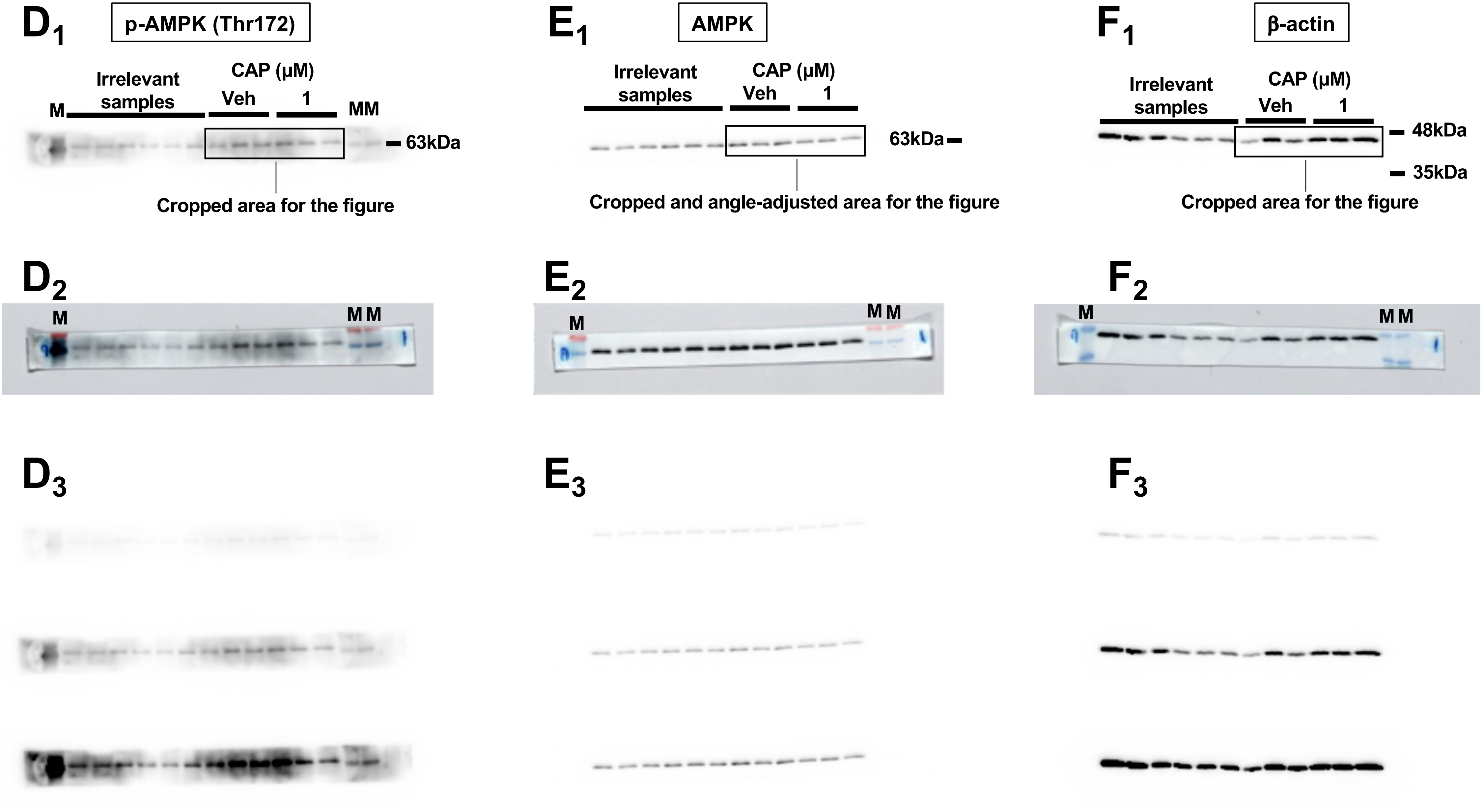
D1
p-AMPK (Thr172)
Irrelevant samples
CAP (µM)
Veh
1
M
MM
63kDa
Cropped area for the figure
E1
AMPK
Irrelevant samples
CAP (µM)
Veh
1
63kDa
Cropped and angle-adjusted area for the figure
F1
β-actin
Irrelevant samples
CAP (µM)
Veh
1
48kDa
35kDa
Cropped area for the figure
D2
M
M M
E2
M
M M
F2
M
M M
D3
E3
F3

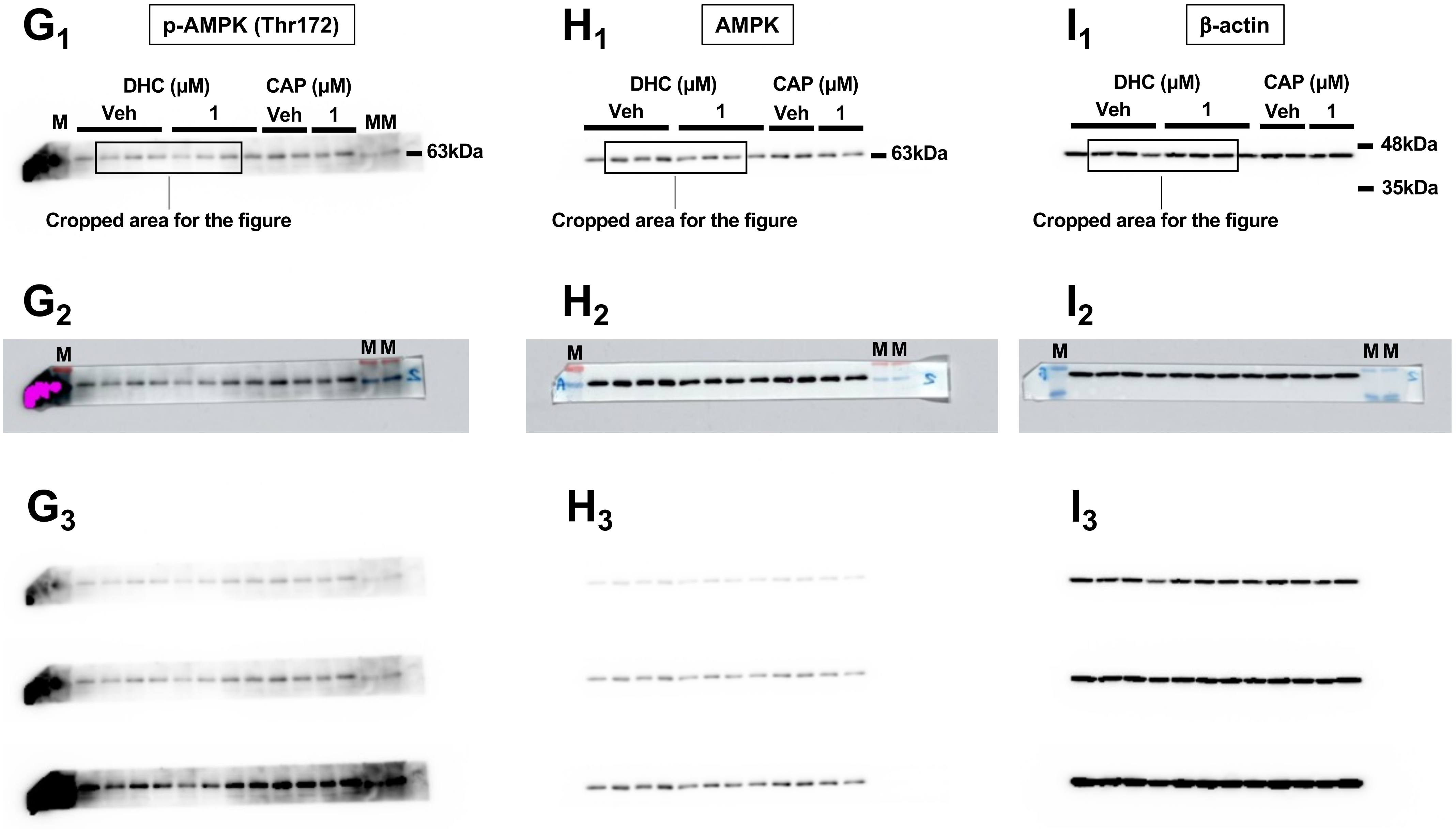
G1
p-AMPK (Thr172)
DHC (μM)
CAP (μM)
M
Veh
1
Veh
1
MM
63kDa
Cropped area for the figure
H1
AMPK
DHC (μM)
CAP (μM)
Veh
1
Veh
1
63kDa
Cropped area for the figure
I1
β-actin
DHC (μM)
CAP (μM)
Veh
1
Veh
1
48kDa
35kDa
Cropped area for the figure
G2
M
M M
H2
M
M M
I2
M
M M
G3
H3
I3

Supplementary Figure 2. The uncropped images of Figure 4a

The membranes were cut into strips that were individually probed with the indicated antibodies. The cropped areas used as the representative pictures in Fig. 4a are enclosed by rectangles. (A_1-C_1) Protein expressions of p-AMPK (A_1), AMPK (B_1) and β-actin (C_1) in 8-MNA-treated 3T3-L1 adipocytes. (D_1-F_1) Protein expressions of p-AMPK (D_1), AMPK (E_1) and β-actin (F_1) in CAP-treated 3T3-L1 adipocytes (including irrelevant samples to this report). (G_1-I_1) Protein expressions of p-AMPK (G_1), AMPK (H_1) and β-actin (I_1) in CAP- or DHC-treated 3T3-L1 adipocytes. (A_2-I_2) The composite pictures generated by ChemiDoc Imager (Bio-Rad, USA) show the edges of the membrane strips. (A_3-I_3) Multiple images taken with various exposure times are shown. M: BLUeye Prestained Protein Ladder marker (Sigma-Aldrich, USA).
